# Practical Notes on Diagnosis and Treatment

**Published:** 1907-06-01

**Authors:** 


### Practical Notes on Diagnosis and Treatment.

**Gall-stones and Cancer.**

It is said that in seven-eighths of the cases of primary cancer of the gall-bladder gall-stones have been present, and it is suggested that the malignant process is due to the irritation set up by the calculi.

**Sodium Benzoate in Pharyngitis, etc.**

Sodium benzoate is of distinct value in the treatment of inflammatory conditions of the upper air-passages—pharyngitis, laryngitis, bronchitis, etc. It may be given in daily doses of 75 grains for a child, 150 grains for an adult.

**Tea in Gout.**

I am a great believer in the value of tea as a preventive of gout, and as aiding in the elimination of uric acid. It should be taken weak and quite freshly made. I also always insist on the avoidance of sweet fruits and on the free use of green vegetables.—*Mr. Jonathan Hutchinson.*

**Gastric Ulcer.**

Dr. George Parker has found calcium chloride of service in cases of gastric ulcer. He quotes two clinical records in which the usual forms of treatment, as by bismuth, peptonised milk, etc., had had very little success, whilst the administration of calcium chloride was followed by prompt relief.

**Optic Atrophy in Disseminated Sclerosis.**

In insular sclerosis optic atrophy is by no means uncommon, but it differs in several respects from that met with in locomotor ataxy. It is less regular and progressive, is often unilateral (at all events for a very long time), and if bilateral, is marked by decided want of symmetry.—*Mr. Lawford.*

**Diarrhœa in Infants.**

The following are useful formulæ to check diarrhœa in infants and young children: (1) Carbolic acid, grs. ij.; bismuth subnitrate, ʒj.; syrup, ℥ss.; water to ℥ij.: a half teasponful every two to four hours. (2) Zinc oxide, grs. j.-ij.; paregoric, ♏. j.-ij.; syrup, ♏. xv.; water to ʒj.: a dose to be given every two or three hours. Thin arrowroot with a few drops of brandy will also help to check diarrhœa.

**Quinine in Pertussis.**

The proper dose of quinine in whooping-cough is $\frac{1}{6}$ grain for each month of a child's age and $1\frac{1}{2}$ grains for each year, given three times in the day—at 6 a.m. and 2 and 10 p.m. More than 6 grains thrice daily is not necessary even for the older children. The remedy should be continued for three weeks and the dose be gradually decreased as the symptoms subside.

**Latent Tuberculosis.**

A saying of Trousseau was, "If you have a case of anæmia which resists treatment by iron, always suspect tubercle or syphilis."

**Chloral Hydrate.**

The taste of this remedy can be masked by ordering the syrup to be taken in half a tumblerful or so of effervescing lemonade.

**Ulcers of the Leg.**

Dr. A. G. Peter confirms Dr. Stephens' recommendation in favour of the administration of calcium iodide in cases of ulcer of the leg. Even instances which had resisted all kinds of treatment for years showed in a week or two clean granulating surfaces, and the induration around the ulcers soon disappeared; in almost all cases complete healing was secured. The dose given was 2 grains thrice daily in a mixture.

**Alkalies in Hyperacidity.**

To relieve the pain of hyperacidity I prefer to order bicarbonate of sodium in doses of 30 to 60 grains dissolved in two ounces of water. The patient is directed to take the first dose two hours after each of the principal meals and to continue every hour until four doses have been taken. Under this treatment the pain after food rapidly diminishes, and finally subsides, pyrosis disappears, and the bowels are moved freely.—*Dr. Soltau Fenwick.*

**Thread Worms.**

For the treatment of thread worms in adults, a condition often very resistant to the ordinary remedies, Dr. A. Newton-Davies advises the following: Two ounces of compound decoction of aloes before breakfast, a diet rich in proteid but of meagre quantity, and a pill (keratin coated) of 2 grains of extract of quassia at bedtime. On the second day a pill is to be given thrice daily, and this is to be continued for a few days, the decoction being repeated when necessary.

**Brachial Neuralgia and Neuritis.**

In these cases it is necessary to enjoin complete and absolute rest (by means of a sling or by bandaging the arm to the side) for the affected limb. The second great factor in treatment is counter-irritation, beginning with mustard leaves and, if necessary, passing on to the thermo-cautery. The counter-irritant should be applied over tender points. Of internal remedies, I have obtained the most satisfactory results with a combination of sodium salicylate with potassium iodide and bromide.—*Dr. Aldren Turner.*

